# Typhoidal Tularemia: 2 Familial Cases

**DOI:** 10.1155/2012/214215

**Published:** 2012-10-17

**Authors:** J. F. Faucher, C. Chirouze, C. Coutris, C. Fery-Blanco, M. Maurin, B. Hoen

**Affiliations:** ^1^Service des Maladies Infectieuses et Tropicales, Hôpital Saint-Jacques, 25030 Besançon Cedex, France; ^2^CNR Francisella Tularensis, Université Joseph Fourier, BP217, 38043 Grenoble, France

## Abstract

Eastern France was not known as a region endemic for tularemia until year 2006. We report on 2 cases of typhoidal tularemia in Eastern France, a 43-year-old hospitalized woman and her husband. Diagnosis was established after fever clearance on serodiagnosis criteria. The source of infection is unclear. As persons in the same family may likely be exposed to a common zoonotic source of infection, tularemia should be considered in the etiologies of familial fever epidemics.

## 1. Introduction

Tularemia belongs to zoonotic diseases. Its recrudescence was recently described in Eastern France [1]. We herein report and review on 2 familial cases of tularemia.

## 2. Case Presentation

A 43-year-old previously healthy female resident of a rural area in Eastern France was admitted in September 2010 for a suspected meningoradiculitis. She had a recent history of fever associated to mild cough, myalgia, and polyarthralgia. Her husband suffered similar flu-like symptoms one day before his wife. Fever started 12 days after a short family stay in Northern Italy. Physical examination results were unremarkable and a chest X-ray was normal. Results of a laboratory work found elevated C-reactive protein (217 mg/L) and liver function tests (alanine amino transferase level: 2 N). An arboviral cause of fever was considered by the GP and she was symptomatically treated. Flu-like symptoms lasted 10 days but epigastralgia and a left hemithoracic pain (T10-T11) appeared with no skin eruption. Laboratory work found persisting C-reactive protein (126 mg/L) and liver function tests (alanine amino transferase level: 2.5 N) elevated values; lipasemia was normal. A gastroscopic examination and a thoracoabdominal CT scan were unremarkable. Two weeks after fever resolution, a meningoradiculitis was suspected because of persisting left hemithoracic pain, and the patient was referred to hospitalization.

A laboratory work up found elevated C-reactive protein (67 mg/L) with normal procalcitonin values. Results of blood and urine cultures were negative. CSF was normal as were the results of a spine RMI. Pregabalin prescription resulted into symptoms improvement. On the basis of serologic results, the following diseases could be ruled out: viral infections (HIV, EBV, CMV, hepatitis B and C, dengue, Chikungunya, Toscana virus, West Nile, TBE), Lyme borreliosis, *Bartonella henselae* infection, rickettsiosis, Q fever, brucellosis, *Legionella*, *Mycoplasma*, *Chlamydia* spp. infections, and yersiniosis. Serum sample was positive for tularemia by the microagglutination (320, threshold of 160) and immunofluorescence assays (IgM: 320; IgG: 640; threshold of 160 for both) [2]. Convalescent serum 3 months later showed a significant decrease of IgM (40) titres while IgG remained stable (640). PCR assays for *Francisella tularensis* (targeting the insertion sequence *ISFtu2* and the gene encoding the surface protein Tul4 [2]) were negative in sera and CSF. The diagnosis of typhoidal tularemia was considered and doxycyclin was started one week after admission. CRP returned to normal 2 weeks afterwards. Her husband who had suffered a similar flu-like syndrome was retrospectively tested for tularemia. His convalescent serum sample was positive at thresholds of 640 (microagglutination), 320 (IgM), and 640 (IgG). None of their 3 children has been sick, and none has been tested.

## 3. Discussion

In both cases, the diagnosis of tularemia was based on serologic assays performed a few weeks after fever clearance. Many differential diagnosis have been ruled out in the hospitalized patient, especially Lyme's disease, in a patient who was admitted initially for a possible meningoradiculitis. As the patient and her husband had suffered a similar flu-like syndrome at the beginning of disease, tularemia was suspected in her husband as well and serologic assays showed evidence of a recent contact with the agent of tularemia.

Although the patients had recently travelled, these 2 cases are probably autochtonous since incubation periods usually range from 2 to 10 days [3], but the patients' exposure was not identified. Eastern France was not known as a region endemic for tularemia until year 2006 [1].

Illness may occur in families or friends because of shared activities or exposures, but very few familial cases have been published [4–8].

Typhoidal tularemia refers to a febrile illness caused by* Francisella tularensis* that is not associated with prominent lymphadenopathy and does not fit into any of the other major forms (ulceroglandular, glandular, oculoglandular, pharyngeal, pnemonic). It may represent less than 10% of tularemia clinical features in Europe [9].

In conclusion, we report for the first time in France on familial cases of typhoidal tularemia. As persons in the same family may be exposed to a common zoonotic source of infection (water, food aerosol), tularemia should be considered in the etiologies of familial fever epidemics.
